# Consolidation of an Olfactory Memory Trace in the Olfactory Bulb Is Required for Learning-Induced Survival of Adult-Born Neurons and Long-Term Memory

**DOI:** 10.1371/journal.pone.0012118

**Published:** 2010-08-13

**Authors:** Florence Kermen, Sébastien Sultan, Joëlle Sacquet, Nathalie Mandairon, Anne Didier

**Affiliations:** Université Lyon 1, Centre National de la Recherche Scientifique, UMR 5020 Neurosciences Sensorielles, Comportement, Cognition, Lyon, France; Centre de Recherches su la Cognition Animale - Centre National de la Recherche Scientifique and Université Paul Sabatier, France

## Abstract

**Background:**

It has recently been proposed that adult-born neurons in the olfactory bulb, whose survival is modulated by learning, support long-term olfactory memory. However, the mechanism used to select which adult-born neurons following learning will participate in the long-term retention of olfactory information is unknown. We addressed this question by investigating the effect of bulbar consolidation of olfactory learning on memory and neurogenesis.

**Methodology/Principal Findings:**

Initially, we used a behavioral ecological approach using adult mice to assess the impact of consolidation on neurogenesis. Using learning paradigms in which consolidation time was varied, we showed that a spaced (across days), but not a massed (within day), learning paradigm increased survival of adult-born neurons and allowed long-term retention of the task. Subsequently, we used a pharmacological approach to block consolidation in the olfactory bulb, consisting in intrabulbar infusion of the protein synthesis inhibitor anisomycin, and found impaired learning and no increase in neurogenesis, while basic olfactory processing and the basal rate of adult-born neuron survival remained unaffected. Taken together these data indicate that survival of adult-born neurons during learning depends on consolidation processes taking place in the olfactory bulb.

**Conclusion/Significance:**

We can thus propose a model in which consolidation processes in the olfactory bulb determine both survival of adult-born neurons and long-term olfactory memory. The finding that adult-born neuron survival during olfactory learning is governed by consolidation in the olfactory bulb strongly argues in favor of a role for bulbar adult-born neurons in supporting olfactory memory.

## Introduction

In mammals, olfactory information is memorized through activation of a combination of cerebral structures, including the olfactory bulb (OB), piriform cortex and, depending on the required task, the hippocampus and amygdala [1]–[6]. Among these brain structures, the OB is known to be the locus of a high level of plasticity linked to memory [7]–[10]. The responses of mitral cells, the relay cells of the OB are modulated by associative learning [11]–[13] as well as by prolonged passive exposure to odors [14]. The oscillatory behavior of the OB is also modulated by learning [15] as is the immediate early gene responsiveness of bulbar interneurons [16]–[19]. The main effectors of plasticity of the bulbar network are thought to be the inhibitory granule cells which regulate output of the olfactory message through reciprocal synapses with the mitral cells [20]. Taken together, these data suggest that the OB has a central role in processing the olfactory signal in relation to its context and significance and so to memorizing it. This is further supported by the fact that inactivation of the OB following associative learning impairs memory retention, suggesting that the OB is involved in consolidation of the memory trace [21]. The cellular mechanisms in the OB involved in memory formation are largely unknown. An NMDA and calcium-dependent synaptic plasticity of the mitral cell response has been reported [22]. Recently, long-term potentiation at the mitral to granule cell synapse has been documented and shown to be supported by adult-born granule cells of the OB [23]. Indeed, the OB contains newborn inhibitory interneurons originating from progenitor cells located in the walls of the lateral ventricles and migrating to the OB where they differentiate mainly into granule cells and to a lesser extent into periglomerular interneurons [24]. The number of newborn granule cells is modulated by olfactory learning through enhancement of their survival rate in the OB [25]–[28]. Furthermore, recent studies have reported long-term memory impairment following reduction of neurogenesis [28], [29], suggesting that adult-born neurons are involved in long-term olfactory memory. This finding is controversial since two other recent studies in which neurogenesis was reduced could not provide any evidence of long-term olfactory memory impairment [30], [31].

Available data thus suggest that through their peculiar physiological properties and increased survival after learning adult-born neurons could play a role in odor long-term memorization. Because the transition from short to long-term memory relies on consolidation processes [32] and because bulbar adult-born neurons may support olfactory long-term memory [28], [29], we propose to investigate the role of consolidation of associative olfactory learning on adult-born neuron survival and long-term memory. To address this issue, we first investigated how a massed learning program occurring over a few hours and allowing no inter-trial consolidation could differentially affect the rate of adult-born neuron survival and memory when compared to a spaced learning program allowing consolidation from one day to the next. Then, to better understand the role of consolidation, we blocked it in the OB using a local infusion of the protein synthesis blocker anisomycin during the spaced training paradigm.

## Results

### Only spaced olfactory learning increased adult-born neuron survival and allowed long-term memory

**Figure 1:**
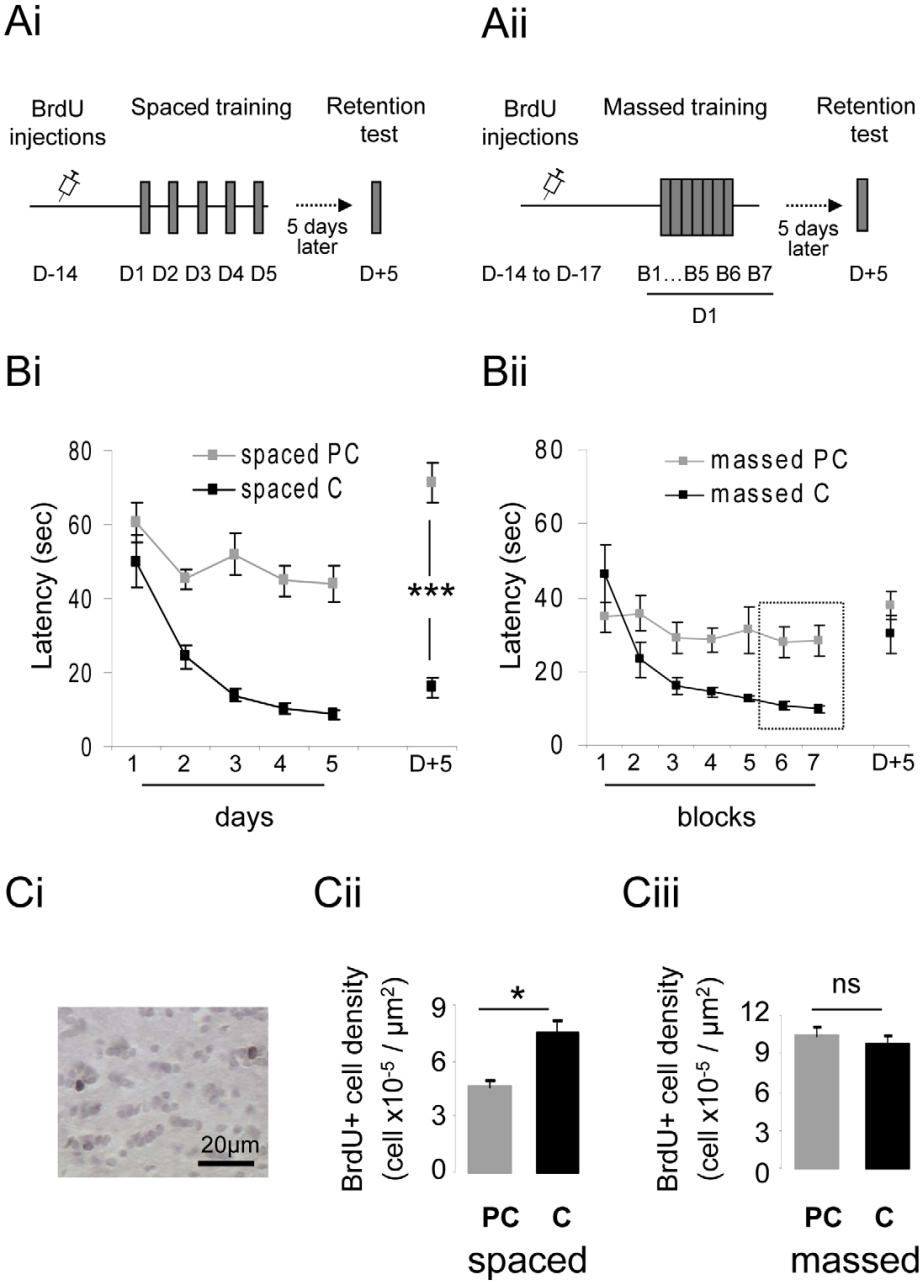
Only spaced (but not massed) learning allowed long-term retention of an associative olfactory task and increased neurogenesis. **A**. Experimental design. For the spaced conditioning (**Ai**) BrdU was injected 14 days prior to training which occurred over 5 days (4 trials per day). For the massed conditioning (**Aii**) BrdU was injected from day 17 to 14 prior to training which occurred during a single day (30 successive trials). In both the spaced and massed training, the mice had to learn to use an olfactory cue to find their reward. 5 days post training, retention of the task was assessed during 4 trials performed under the same conditions as during the learning phase. **B**. Behavioral performance was assessed by measuring the latency to find the reward in groups of conditioned (C) and pseudo-conditioned animals (PC) where the odor was pseudo-randomly associated with the reward. In the spaced training paradigm, latency decreased in the conditioned but not the pseudo-conditioned mice indicating that the conditioned animals had learned the task. 5 days post-training, the conditioned mice remembered the task (C versus PC, bilateral t-test, p<0.001) (**Bi**). In the massed training paradigm, the performance of the conditioned animals decreased from block 1 to block 7 (4 trials per block for B1 to B5 and 5 trials per block for B6 and B7) and differed from that of pseudo-conditioned animals. Massed conditioned animals did not remember the task 5 days post training (**Bii**). **C**. Adult-born cell counts. The density of BrdU-positive cells (**Ci**) in the granule cell layer of the OB was increased in the conditioned animals of the spaced group (**Cii**) but not the massed trained animals (**Ciii**) compared to their respective pseudo-conditioned groups. *:p<0.05; ***:p<0.001; ns: non-significant (p>0.05).

In order to assess the effect of consolidation on adult-born neuron survival and long-term retention, we compared two associative learning paradigms in which the inter-trial interval (ITI) was varied in order to facilitate or hamper the consolidation process. We submitted two groups of young adult mice to an associative olfactory learning program using +limonene reinforced by a food reward (see Methods). The first group underwent spaced learning consisting in sessions of 4 trials per day (ITI = 15 min) over five days (total of 20 trials) (Figure 1Ai). A second group underwent massed learning consisting in trials (ITI = 15 min) performed over the same day (Figure 1Aii). Both groups were assessed for long-term memory of the task by a retention test 5 days post-training. Control groups were pseudo-conditioned (reward randomly associated with the odorant, see Methods) for each learning paradigm. In the spaced group, conditioned animals performed differently from pseudo-conditioned animals (group effect; F(1,17) = 33.9, p<0.001) and learning was effective as shown by the significant decrease of latency to find the reward (day effect, F(4,40) = 21.96, p<0.001) (Figure 1Bi) observed in the conditioned animals. In contrast, in the pseudo-conditioned animals, latency remained stable throughout the training period indicative of no learning (day effect, F(4,45) = 1.929, p>0.05) (Figure 1Bi).

In the massed group, latency was calculated for blocks of four trials for easier comparison with the spaced training (blocks 1 to 5 on Figure 1Aii). After 20 trials, the animals showed a reduction in latency (block effect, F(4,45) = 10.62, p<0.01) (Figure 1Bii) and performed differently from the pseudo-conditioned animals (group effect, F(1,23) = 5.57, p<0.05) in which latency did not decrease with blocks (block effect, F(6,98) = 1.94, p>0.05). However, latency was higher than in the spaced group (p = 0.006 for difference between block 5 in the massed group and day 5 in the spaced group), indicating a difference in performance between the groups after 20 trials (Figure 1B). The conditioned animals in the massed group had not learnt the task as well as had the spaced group.

In an attempt to obtain the same level of performance in the massed group as in the spaced group, the mice in this massed group performed a further 10 trials (represented as two blocks of five trials, blocks 6 and 7 on Figures 1Aii and Bii). After a total of 30 trials, their latency to find the reward was similar to that observed in the spaced group after only 20 trials (p>0.05 for difference between block 7 in the massed group and day 5 in the spaced group) (Figure 1Bii). Using the success rate as another index of learning, we also found that both spaced and massed conditioned groups performed differently from their control groups (group effect; pseudo conditioned versus conditioned groups; spaced group F(1,17) = 24.49, p<0.001; massed group F(1,23) = 7.0, p<0.05), learned the task (day effect; F(4,40) = 7.5, p<0.001 for spaced conditioned group; block effect; F(6,63) = 3.6, p<0.01 for massed conditioned group) and reached similar levels of performance (spaced conditioned versus massed conditioned group at the end of training: bilateral t-test, p>0.05).

Taken together, these results indicate that at the end of the complete training period, the mice in both conditioned groups had learnt the association between odor and reward and, even if the massed group needed more trials, it was able to attain similar performance levels at the end of training as the spaced group.

Subsequently, long-term retention of learning was assessed 5 days (D+5) after the training period for both the spaced and massed groups (Figure 1Ai, Aii, Bi and Bii). The spaced-trained animals clearly remembered the odor-reward association; their mean latency values were lower than the pre-training levels (p<0.001) (Figure 1Bi), and they performed better than the pseudo-conditioned group (p<0.001). In contrast, we found that the massed-trained mice did not remember the odor-reward association 5 days after conditioning; the mean latency values returned to pre-training levels (p>0.05) and were similar to those of the pseudo-conditioned group (p>0.05) (Figure 1Bii). In both pseudo-conditioned groups which did not learn the task, latency on D+5 was similar to that of pre-training level (p>0.05 in massed and spaced pseudo-conditioned groups). The same results were obtained using the success rate as an index of performance. In conclusion, spaced-trained animals remembered the association 5 days after the end of acquisition whereas massed-trained animals did not. Since performance levels at the end of training were similar in both groups, this finding indicates that consolidation of the acquired memory trace during the 24-hour interval separating the training sessions in the spaced paradigm was necessary for long-term retention of the task.

**Figure 2:**
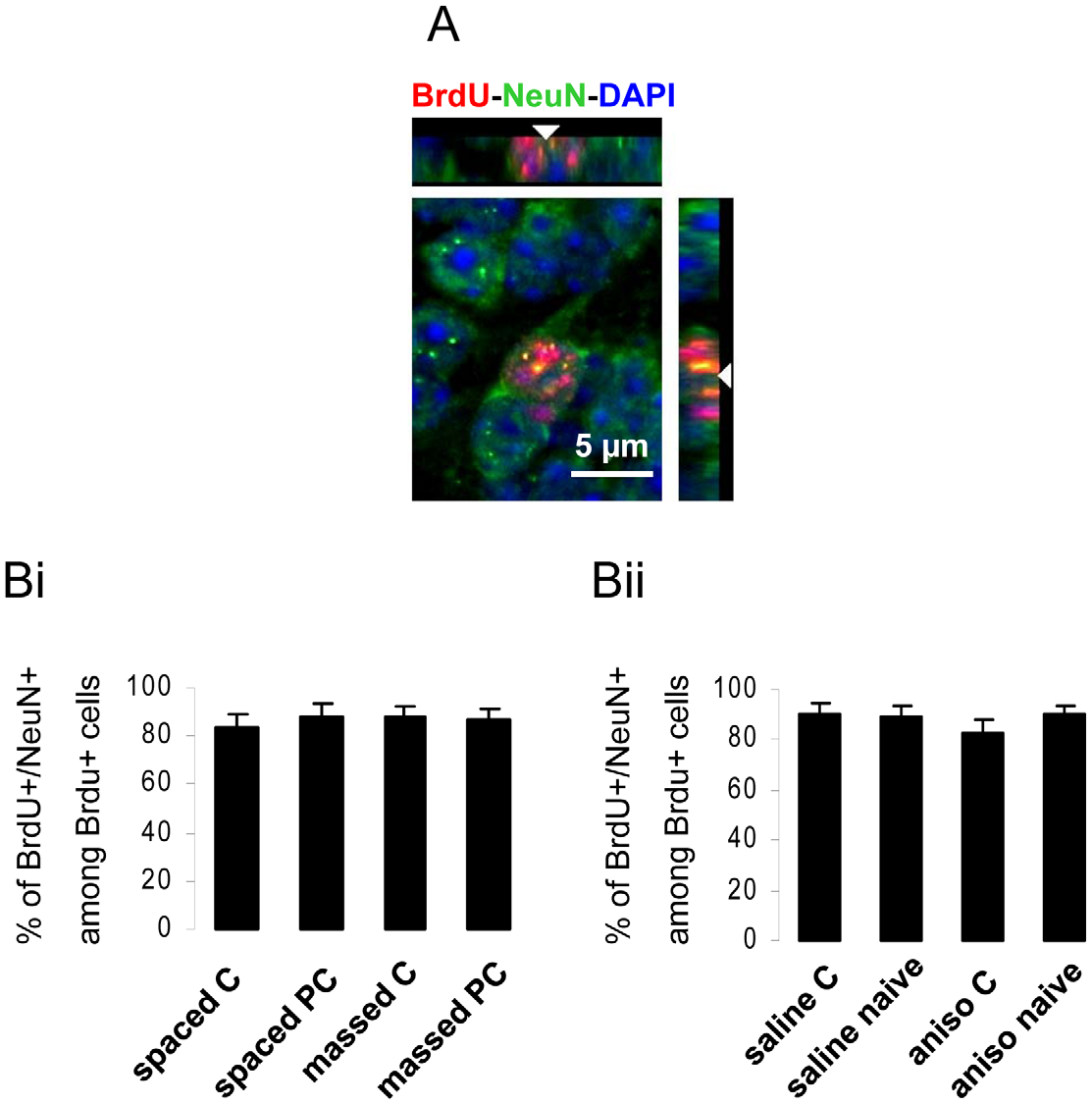
Neuronal differentiation in conditioned (C), pseudo-conditioned (PC) and naive animals. **A**. Representative BrdU/NeuN double-labeled cell with orthogonal views. **B**. No difference in percentage of double-labeled cells was found between spaced and massed trained animals (group effect, F(3,8) = 0.231, p = 0.87) (**Bi**), nor between saline and anisomycin injected animals (group effect, F(3,5) = 0.512, p = 0.69) (**Bii**).

We then looked at the effect of these two different learning paradigms on the rate of neurogenesis in the OB, known to be affected by learning [26], [28]. Due to the Bromodeoxyurine (BrdU) injection protocol, changes in the rate of neurogenesis will reflect modulation of the adult-born cell survival. The density of newborn cells (Figure 1Ci) was then assessed in the granule cell layer of both groups (see Methods). Following spaced conditioning, the density of newborn cells was increased compared to that of the pseudo-conditioned animals (p<0.05) (Figure 1Cii). Interestingly, the massed conditioning did not modulate neurogenesis; the level of newborn cells was similar in the conditioned and pseudo-conditioned animals (p>0.05) (Figure 1Ciii). The difference in BrdU-positive cell density in the massed versus spaced groups is due to the difference in the number of BrdU injections (see Methods). The percentage of BrdU-positive cells expressing the neuronal marker NeuN (≈85%) was similar in all groups (Figure 2A, Bi). These data indicate that the 24-hour time interval was necessary to allow a learning-induced increase in neurogenesis and thus that newborn neuron survival may be related to memory consolidation processes.

### Protein synthesis-dependent mechanisms occur in the OB and are required for increased survival of newborn cells and long-term olfactory memory

Because the OB network is known to be modulated by learning and involved in post-learning mechanisms, we further tested the dual hypothesis that consolidation may occur in the OB and so support the long-term retention and that consolidation is required for the neurogenic effect of learning. To do this we used intra bulbar anisomycin to block consolidation [33], [34] in the OB and looked first at memory performance and then at the level of neurogenesis.

**Figure 3:**
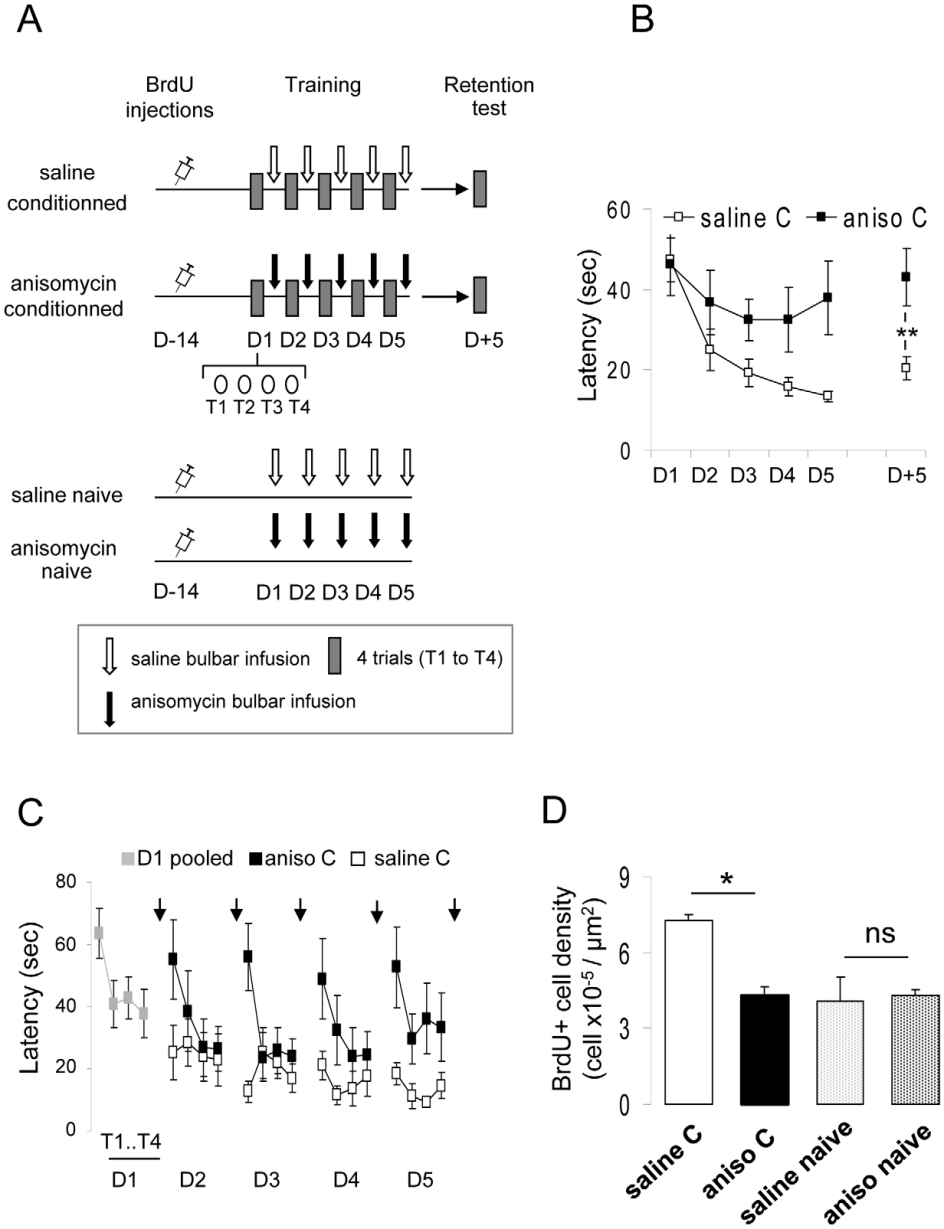
Anisomycin infusion in the olfactory bulb during the spaced learning blocked improvements in performance and increase in neurogenesis. **A**. Experimental design. BrdU was injected 14 days before training. Animals underwent spaced olfactory associative learning and were infused after each training session with either anisomycin or saline. Naive untrained animals were similarly infused with anisomycin or saline. A retention test was performed 5 days post-training. **B**. Behavioral performance. Conditioned saline-infused (saline C) animals learned the task as shown by the decrease in latency and remembered it after 5 days. In contrast, conditioned anisomycin-infused (aniso C) animals did not show any change in latency. **C**. Trial by trial analysis of the learning curve further showed that anisomycin-infused animals returned to pre-training performance between each training session in contrast to saline-infused animals. However, they showed within-session learning. Black arrows symbolize post-training bulbar infusions. **D**. Adult-born cell counts. Conditioning increased BrdU-positive cell density in saline-infused animals. The infusion of anisomycin prevented this effect without affecting the basal rate of neurogenesis. *: p<0.05; **: p<0.01; ns: non-significant (p>0.05).

Animals were trained using the spaced paradigm, as for the first experiment. Immediately after the first training session, the mice were divided into two groups; one was infused in the OB with anisomycin (2 µL per OB, 100 µg/µL) and the other with saline. Intra-bulbar infusions were performed 10 min after the end of each training session (Figure 3A). Performances in the anisomycin- and saline-treated groups were different (group effect, F(1,17) = 4.592, p<0.05). We found that the behavioral task was rapidly acquired in the saline-injected animals as evidenced by the decrease in latency with time (day effect, F(4,45) = 12.842, p<0.001) (Figure 3B) and the increase in their success rate (day effect, F(4,45) = 11.69, p<0.001). However in the anisomycin-infused animals, no change in latency (day effect, F(4,40) = 0.532, p>0.05) (Figure 3B) or in success rate (day effect, F(4,40) = 2.38, p>0.05) was observed indicating that the protein synthesis blocker altered learning of the associative olfactory task.

To analyze the learning process in anisomycin versus saline-treated mice in more detail we looked at the evolution of latency across trials during one conditioning session (4 successive trials per day of training; Figure 3C). In the saline-infused mice, latency on the first trial of each session decreased each day (day effect for the first trial of each session, F(4,45) = 12.262, p<0.0001) whereas in the anisomycin-treated mice the latency of the first trial each day was identical to the pre-training level (day effect for the first trial of each session, F(4,40) = 0.071, p>0.05) showing that they forgot the task from one day to the next. Finally, as in the first experiment, the saline-infused animals still remembered the task 5 days later as shown by latency values that remained lower than the pre-training levels (p<0.001), while the latency values of anisomycin-treated mice stayed at the same level as during training (p>0.05) (Figure 3C).

**Figure 4:**
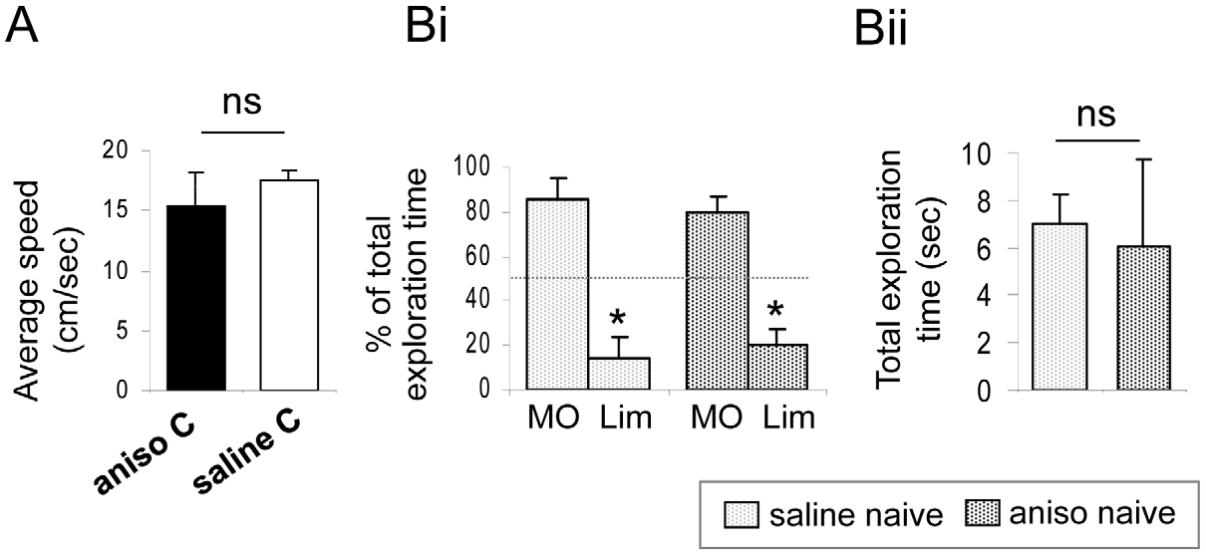
Anisomycin infusion did not alter locomotion, odor detection or exploratory behavior. **A**. The videotracking assessment of locomotion on Day 5 of conditioning showed no difference between the saline- and anisomycin-infused animals. **B**. Spontaneous exploratory behavior of an odorized (+limonene: Lim) versus a non- odorized hole (mineral oil: MO) was measured during a non-reinforced trial in naive untrained animals. Animals differentially explored the two holes, indicating that they detected the odor. In this case, they spontaneously avoided the odorized hole. **C**. The total time spent by the animals exploring the two holes was also recorded as an index of their exploratory behavior and showed no difference between groups. *: p<0.05; ns: non-significant (p>0.05).

Importantly, daily anisomycin injections did not affect basic olfactory sensory and mnesic processes since the animals showed within-session improvements in performance, indicating that their ability to learn the task across successive trials was intact (within-session trial effect on Day 2 to Day 5, F(3,128) = 5.959, p = 0.001, Figure 3C). Furthermore, differences in learning observed between anisomycin- and saline-treated mice could not be attributed to differences in locomotor's activity which was recorded on Day 5 (see Materials and Methods) and showed no difference between groups (p>0.05) (Figure 4A). Moreover, the effect of treatment on olfactory detection was assessed on Day 5 in naive animals treated with saline or anisomycin. The proportion of time spent investigating an odorized versus a non-odorized hole on the board was similar in both groups (one sample *t*-test for difference from 50%; saline naive: p<0.05, anisomycin naive p<0.05) (Figure 4Bi) showing no impairment in odor detection in the anisomycin-treated mice. The total time spent investigating the holes was also similar in both groups (p>0.05) (Figure 4Bii) indicating that the anisomycin infusion did not alter exploratory behavior.

In summary, local bulbar anisomycin treatment prevented consolidation of olfactory memory from one day to the next but did not alter either basic odor processing or within-session learning. These results demonstrate that the OB is central for consolidation processing of an associative olfactory memory trace.

The density of newborn cells was then assessed in the granule cell layer of anisomycin and saline-infused animals. As expected, in the saline-infused animals, olfactory conditioning increased the density of adult-born cells compared to that in non-conditioned animals (p>0.05) (Figure 3D). However, animals daily infused with anisomycin were not able to learn the task and presented a level of neurogenesis lower than did the saline-infused conditioned animals (p<0.05) (Figure 3D).

It is important to note that the density of newborn cells was similar between the naive (non-conditioned) animals infused with either saline or anisomycin (Figure 3D), indicating that anisomycin by itself was not altering the survival of newborn neurons in the OB. This finding indicated that anisomycin alone, in the absence of memory consolidation demands did not affect adult-born neuron survival. The percentage of BrdU-positive cells which expressed the neuronal marker NeuN (≈85%) was similar in all groups (Figure 2A, Bii).

Taken together, the results show that consolidation in the OB is required to increase learning-induced neurogenesis and long-term retention of a task.

## Discussion

The present results show that neurogenesis was increased in the OB of the group which underwent the spaced training, when there was sufficient time for consolidation to occur. The consolidation occurring during the massed training was not sufficient to allow increased survival of adult-born neurons. Two approaches led us to this conclusion. First, behavioral manipulation of the ITI showed that when more time was allowed for consolidation (spaced learning) then better long-term retention of the task was observed together with an increased survival rate of adult-born neurons in the OB. These two effects were not observed in the massed learning protocol. Consistent with performances observed in spatial learning during spaced or massed training [35], we found that less trials were required for learning acquisition during a spaced versus a massed paradigm. Secondly, when the consolidation processes in the OB were pharmacologically blocked using the protein synthesis inhibitor anisomycin [36], then the spaced learning failed to promote long-term retention of the task and enhanced neurogenesis. Locally infusing anisomycin into the OB caused no observable side effects in that the treated animals were able to perform basic processing of an olfactory cue and showed no alteration in their locomotive or exploratory behavior. The results of these two experiments thus indicate first that the consolidation of olfactory learning in the OB is required for long-term retention and second, that it also determines survival of adult-born neurons. Although consolidation processes may occur in several other structures of the olfactory memory network [37]–[39], our findings clearly show that consolidation in the OB is essential to long-term memory formation. Regarding neurogenesis, this finding has several important implications. It is known that many newborn neurons die upon their arrival in the OB [40], [41] and that they can be saved by learning [25]–[28]. Since a blockade of consolidation in the OB prevented learning-induced enhancement of neurogenesis, our data provide evidence that the mechanism determining adult-born neuron survival is linked to consolidation. Given the wide spectrum of the cellular mechanisms potentially affected by anisomycin, it is important to note that this drug showed no observable toxic effect on newborn cells. Indeed, naïve anisomycin-infused animals showed the same level of neurogenesis as did saline-infused animals. The lack of enhancement of neurogenesis in the anisomycin-infused animals can thus be ascribed to a consolidation blockade. We found that the blockade of consolidation affected neurogenesis and memory, raising the issue of a link between neurogenesis and memory. It cannot be excluded that anisomycin affects memory-related processes other than neurogenesis. However, our results also showed that the enhancement of adult-born neuron survival was closely correlated to retention of the task. This confirmed recent findings showing that adult-born neurons surviving after learning are functionally integrated into the bulbar network responding to the learned odor and are essential to long-term retention of an associative olfactory task [28], [29].

At the cellular level, long-term potentiation has been reported in newborn neurons [23] and could initiate molecular events [42] preventing the cell death of those newborn neurons involved in processing the learned odor. Adult-born neurons would thus be tagged during learning and specifically selected by post-learning processes as suggested in the dentate gyrus of the hippocampus [43]. Based on these data, we suggest that protein synthesis enables newborn neuron survival during learning and these newborn neurons allow long-term memory. A consolidated long-term olfactory memory would thus require this two-step process. This conclusion is in line with our previous finding that newborn neurons support long-term memory [28].

While there is abundant literature using anisomycin on consolidation processes occurring in many brain regions involved in different forms of learning [33], [34], [44], [45], little data are available for the olfactory system [37], [46]–[49] and what there is do not deal with the role of the OB. Our data indicate that the OB plays an essential role in consolidation of the memory trace, thus delivering a consolidated representation of the learned odor to the higher olfactory structures [38], [50]. This is in agreement with previous work showing that post-learning local bulbar anesthesia altered the long-term memory of an associative olfactory task [21] and that systemic injection of anisomycin prevented consolidation of a learned odorant in newborn rabbit [51].

In conclusion, memory consolidation processes in the OB determine adult-born neuron survival suggesting that neurogenesis modulation is a key effector of olfactory memory.

## Materials and Methods

### Animals

73 adult male C57Bl/6J mice (Charles River, L'Arbresles, France) aged 8 weeks at the beginning of the experiment were used. Every effort was made to minimize the number of animals used and their suffering during the experimental procedure. In accordance with the policy of Lyon1 university and the french legislation, experiments were done in compliance with the European Community Council Directive of November 24,1986 (86/609/EEC), and those of the French Ethical Committee.

44 mice were involved in the spaced versus massed experiment (spaced conditioning n = 9; massed conditioning n = 10; spaced pseudo-conditioning n = 15; massed pseudo-conditioning n = 10).

A second cohort of 29 mice was used in the second experiment: 19 mice underwent the spaced olfactory conditioning (n = 9 infused with anisomycin and n = 10 infused with saline) and 10 mice were used as controls and were not conditioned (n = 5 infused with anisomycin and n = 5 infused with saline).

All behavioral training was conducted in the afternoon. In the massed training, the duration of the behavioral experiment was longer than in the spaced training (more trials per day) and the behavioral learning session thus began 2 hours earlier. Mice had free access to water and food except during the olfactory learning period when they were maintained on a food-deprivation schedule designed to keep them between 85–95% of their body weight over the behavioral testing period.

### Behavioral experiment

#### Experimental set-up

The mice were tested using an automated computer-assisted 2-hole board apparatus (40 cm×40 cm) [52]. The trial started by placing the mouse on the board facing the holes (3 cm diameter, 4.5 cm deep), and latency (time to find the reward) was recorded. The holes contained a polypropylene swab impregnated with +limonene (purity>97%, Sigma-Aldrich, Saint Louis, MO, USA) or mineral oil.

#### Shaping

During the shaping (3 days, 4 trials per day, inter-trial interval, ITI = 15 min) the mice were allowed to dig through the bedding for 90 sec to retrieve a reward (a small piece of sweetened cereal, Kelloggs, Battle Creek, MI). During the first few trials, the reward was placed on the top of the bedding of one of the two holes. After several successful retrievals, the reward was buried deeper into the bedding. Shaping was considered to be complete when a mouse could successfully retrieve a reward that was deeply buried in the bedding. No odorant was used for this task.

#### Spaced learning

This was performed over 5 days (4 consecutive trials/day, ITI = 15 min, 90 sec per trial). One of the two holes was odorized with +limonene (20 µL of pure odorant). The reward was systematically buried in the odorized hole, whereas the non-odorized hole contained no reward. Once the mice found the reward, they were allowed to eat it and returned to their home cage until the next trial. The position of the reinforced hole was randomized to prevent any spatial learning. Additional mice were used for the pseudo-conditioned group, in which the reward was pseudo-randomly buried either in the odorized hole or in the non-odorized hole. The randomization was controlled in order to avoid 3 trials in a row with the reward in the same hole.

#### Massed learning

In this group, the mice underwent the same conditioning procedure as for the spaced learning except that all the trials were carried out on the same day (30 consecutive trials, ITI = 15 min, 90 sec per trial). Additional mice were used for the pseudo-conditioned group.

#### Retention test

Five days after the final conditioning session, the animals had to perform 4 trials on the board in exactly the same conditions as during their learning period.

#### Data analysis

For each trial, latency to find the reward was recorded as an index of learning [25], [28]. Success rate was also calculated for each session, a successful trial being recorded when the animal first visited (nose poking) the odorized hole. For each behavioral session, mean latencies and success rates were calculated and averaged within groups. The figures show the results as mean ± sem. Between-groups comparisons were done using ANOVA for repeated measures (Systat software, SSI, Richmond, CA, USA) and by unpaired or when applicable by paired bilateral *t*-tests. Statistical significance was set at p<0.05.

### Drug administration in the OB

#### Surgery

Prior to surgery, the mice were anesthetized with a cocktail injection of 50 mg/kg ketamine and 7.5 mg/kg xylazine (i.p.) and secured in a stereotaxic instrument (Narishige Scientific Instruments, Tokyo, Japan). All the animals were implanted with double guide cannulae (26-gauge; Plastics One Roanoke, VA, USA) located just above both OBs at the following coordinates with respect to the bregma: AP, +5 mm; ML, ±1.5 mm; DV, −1 mm. The tips of the guide cannulae were positioned 1 mm dorsal to the target infusion site; consequently, the infusion cannulae extended 1 mm from the end of the guide cannulae and were positioned to be in the middle of the OBs. Four screws were drilled into the skull, and dental cement was used to secure the guide cannulae and cover the incision area. Dummy infusion cannulae were then placed into the guide cannulae to prevent blockage or infection. The mice were allowed 12 days to recover from the surgery in individual cages with food and water *ad libitum* prior to BrdU administration.

#### Infusion procedure

The protein synthesis inhibitor anisomycin (Sigma, France) was used to block consolidation in the OB (100 µg/µL in 0.9% NaCl, pH = 7.4) [37], [53]. 10 µL Hamilton syringes containing either anisomycin or saline were attached to the cannulae with a polyethylene tube and driven by an infusion pump (Harvard Pump). Drugs were delivered bilaterally into awake or anesthetized mice when needed (isoflurane, 2 min induction at 4.5%; 2 min at 2.5%) at a rate of 0.4 µL/min for 5 min (2 µL total volume delivered per side). The position of the cannulae and the infusion volume had been checked previously using 2% pontamine sky blue dye solution in saline; this proved that a 2 µL infusion was distributed adequately throughout the OB without appreciable invasion of other neural structures [54]. The infusion cannulae were left in place for one minute after the infusion ended in order to minimize backflow. A drug or saline solution was infused each day, 5 min after the end of the last trial. To assess the effect of the anisomycin or saline injections on bulbar neurogenesis, additional control mice were injected under exactly the same conditions except that the animals had not been conditioned in any way.

#### Locomotor activity

In order to assess the potential side effects of these intra-cerebral drug injections, each mouse's movement on the hole-board was recorded at Day 5 using a video camera (ViewPoint, Champagne au Mont d'Or, France). Each mouse's average locomotion speed was then calculated and compared between groups using bilateral t-tests.

#### Odor detection test

To determine whether the injected drug would affect olfactory detection, the mice were placed on the two-hole board apparatus for 2 min. One of the two holes was odorized with +limonene (20 µL of pure odorant), and the other contained mineral oil. The time spent exploring the holes was recorded. Time spent investigating the odorized hole differed from chance level (one-sample t-test for difference from 50%) indicating odor detection. In addition, to ensure that the exploratory behavior was not affected by the intra-cerebral drug infusions, the total time spent visiting the two holes was also measured.

### Assessment of neurogenesis

#### 5-Bromo-2′-deoxyuridine (BrdU) administration

Previous studies have shown that adult-born neurons are particularly sensitive to olfactory activity during a critical period starting 2 weeks after their birth [55], [56]. This interval corresponds to the time required for these newborn cells to migrate from the sub ventricular zone to the OB [41]. To have a cohort of 2-week old labeled cells in the OB of mice in the spaced group at start of learning, we injected the Bromodeoxyurine 14 days before the first day of conditioning (3 injections, 2 h interval, 50 mg/kg (Figure 1Ai). Using this protocol, BrdU-labeled cells present in the OB during learning were aged 14 to 18 days. To obtain labeled cells of similar age in the massed group, these mice were first injected with BrdU 17 days before conditioning began and then for the following 4 days (2 injections per day, 50mg/kg) (Figure 1Aii). In this manner, in massed-trained animals, BrdU labeled cells present in the OB during the day of training would also be aged between 14 and 17 days.

All animals were sacrificed 5 days post training, one hour after the end of the retention test.

#### BrdU immunocytochemistry

Mice randomly taken from each experimental group (n = 3–5 per group) were deeply anesthetized (Pentobarbital, 0.2 mL/30 g) and killed by intracardiac perfusion of 50 ml of fixative (4% paraformaldehyde in phosphate saline buffer, pH 7.4). Their brains were removed, post-fixed, cryoprotected in sucrose (20%), frozen rapidly and then stored at −20°C before sectioning with a cryostat (Microtech). Brain sections were first incubated in Target Retrieval Solution (Dako, Trappes, France) for 20 min at 98°C. After cooling for 20 min, they were treated with Triton 0.5% (SigmaX100) in phosphate buffered saline (PBS) for 30 min then for 3 min with pepsin (0.43 U/ml in 0.1N HCl, Sigma). Endogenous peroxidases were blocked with a solution of 3% H_2_O_2_ in 0.1 M PBS. Sections were then incubated for 90 min in 5% normal horse serum (Vector Laboratories,Burlingame, CA, USA) in 5% bovine serum albumin (BSA, Sigma) and 0.125%Triton X-100 to block non-specific binding, and then incubated overnight at 4°C in a mouse anti-BrdU primary antibody (1/100, Chemicon, Temecula, CA). They were then incubated in a horse biotinylated antimouse secondary antibody (1/200, Vector) for 2 h and processed with avidin-biotin-peroxydase complex (ABC Elite Kit, Vector) for 30 min. Finally the sections were reacted in 0.05% 3,3′-diaminobenzidine-tetra-hydrochloride (DAB,Sigma), 0.03% NiCl_2_ and 0.03% H_2_O_2_ in Tris–HCl buffer (0.05 M, pH 7.6), dehydrated in graded ethanols, and coverslipped in DPX.

#### BrdU-positive cell quantification

All cell counts were conducted blind with regards to mouse status. Data were collected using mapping software (Mercator Pro, Explora Nova, La Rochelle, France), coupled with a Zeiss microscope. For each mouse, BrdU-positive cells were counted on 20 consecutive sections (14 µm thick, 70 µm intervals) from the granule cell layer of the right OB. Cell density (number of labeled profiles/µm^2^) was calculated for each section and averaged for each animal and then averaged across animals within each group. Between-groups comparisons were performed using bilateral Student's *t*-tests.

#### Double-labeling analysis

To determine the phenotype of the BrdU-positive cells in the OB, BrdU/NeuN double-labeling was performed using rat anti-BrdU (1∶100, Harlan Sera lab, Loughborough, UK) and mouse anti-NeuN (1∶500, Chemicon). The appropriate secondary antibodies coupled to Alexa 546 (Molecular Probes) to reveal BrdU and Alexa 488 (Molecular Probes) to reveal NeuN were used. BrdU-positive cells were examined for co-labeling with NeuN (10–20 cells per animal, n = 2–3 animals per group). The double-labeled cells were observed and analyzed by pseudo-confocal scanning microscopy using a Zeiss microscope equipped with the ApoTome system. The percentage of double-labeled cells was calculated for each group and compared using ANOVA followed by Bonferroni *post hoc* tests.
